# Detection of Interference Phase by Digital Computation of Quadrature Signals in Homodyne Laser Interferometry

**DOI:** 10.3390/sl21014095

**Published:** 2012-10-19

**Authors:** Simon Rerucha, Zdenek Buchta, Martin Sarbort, Josef Lazar, Ondrej Cip

**Affiliations:** Institute of Scientific Instruments of the ASCR, Kralovopolska 147, 612 64 Brno, Czech Republic; E-Mails: buchta@isibrno.cz (Z.B.); martins@isibrno.cz (M.S.); joe@isibrno.cz (J.L.); ocip@isibrno.cz (O.C.)

**Keywords:** laser interferometry, optical metrology, homodyne detection, digital signal processing

## Abstract

We have proposed an approach to the interference phase extraction in the homodyne laser interferometry. The method employs a series of computational steps to reconstruct the signals for quadrature detection from an interference signal from a non-polarising interferometer sampled by a simple photodetector. The complexity trade-off is the use of laser beam with frequency modulation capability. It is analytically derived and its validity and performance is experimentally verified. The method has proven to be a feasible alternative for the traditional homodyne detection since it performs with comparable accuracy, especially where the optical setup complexity is principal issue and the modulation of laser beam is not a heavy burden (e.g., in multi-axis sensor or laser diode based systems).

## 1. Introduction

The laser interferometry represents the most precise technique for measurement of geometrical quantities today. Among currently known methods, it provides the best resolution and accuracy and ultimate (theoretically an infinite) dynamic range. The basic resolution of the laser interferometer corresponds to the wavelength of the laser source, but for many applications such an accuracy is insufficient. The resolution is further improved by various methods, commonly referred to as fringe subdivision or detection techniques [1,2].

Generally speaking, the detection methods usually expand the basic concept of interferometric measurement by adding extra information to the interference signal that helps to extract the phase information with an increased precision [3,4]. A typical example is the homodyne detection that handles the polarisation of the laser beam in the optical hardware so that a compound wave is produced instead of the interference signal. The phase information is then extracted using additional electro-optic system that produces a pair of interference signals in quadrature (see details in the next section). The phase is then extracted from these signals with significantly increased precision.

In this paper, we present a novel detection technique, related to a traditional homodyne detection, that relies on a digital computation instead of rather complex optical processing. It employs a frequency modulated laser source on the input of a non-polarising laser interferometer and a simple photodetector that observes the output. Then a combination of synchronous demodulation, linear transforms, mixing, phase shifting and trigonometric transforms is used to reconstruct the same pair of quadrature signals. The detection is further improved by a scale linearization techniques that mitigate the measurement error introduced when the method is applied in a real measurement system.

The presented approach brings several advantages, especially reduced demands on the precise construction, reliability and cost of laser interferometry based measurement systems. Our aim was not to present a novel technique that would allow for better precision, accuracy or bandwidth—especially the bandwidth is limited by the frequency modulation bandwidth of the laser source (we deal with DC—100 Hz in our experimentation). Instead, we proposed a method that achieves a performance comparable to that of the current methods, *i.e.*, decent resolution (sub-nanometre).

The rest of this paper is organised as follows. Section 2 summarises the principles of the homodyne detection and presents an analytic description of the proposed method. Section 3 describes the experiments we have carried out to validate the performance of our method in comparison with a homodyne detection, while Section 4 presents the results and holds the discussion. Finally, Section 5 summarises and concludes the paper.

## 2. Methods

### 2.1. Homodyne Detection with Polarising Optics and Quadrature Detection

**Figure 1.**
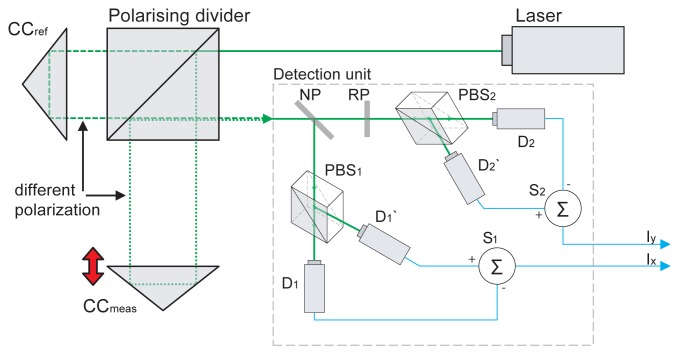
Polarising laser interferometer with quadrature detection unit for homodyne detection: the interferometer employs the polarising divider, the reference corner cube reflector CC*_ref_* and measurement reflector CC*_meas_*. The output beam is compound from two beams with a different polarisation. The detection unit splits the beam into two detection branches on the non-polarising divider NP In the first branch, the polarising beam splitter PBS1 adjusts the polarisation to a single plane so that the interference occurs and is observed by photodetectors D1, D1′ (mutually in an opposite phase). In the second branch, one of the wave components is retarded by the retarder plate RP The photodetectors D2, D2′ observe the interference that occurs due to rotation of the polarisation plane on the polarising beam splitter PBS2. The subtracter SI produces the signal *I_x_* as a difference between signals D1 and D1′, the subtracter S2 produces *I_y_* from D2, D2′ in the same way.

In a typical Michelson setup with homodyne detection, the interferometer setup contains a polarising beam splitter to produce the measurement and reference beams with a different polarisation plane. As a result, there is a complex light beam compound from the waves with mutually perpendicular polarisation on the output of the interferometer rather than a simple interference. That compound beam is further processed by an optoelectronic detection system, usually referred to as a quadrature detection unit (schematically shown in Figure 1), that produces a pair of interference signals *I_x_* and *I_y_* that are in quadrature, *i.e.*, mutually phase shifted by *π*/2.

The immediate value of the interference phase *φ*(*t*) is extracted by a quadrature detection, *i.e.*, the interference phase is calculated using an inverse function

(1) $$\phi (t)=\arctan\left(\frac{I_{y}(t)}{I_{x}(t)}\right)$$

from immediate values of the X- and Y-axis signals *I_x_* and *I_y_*.

One of our objectives was to propose a method that would be simpler in terms of optical hardware complexity. This complexity is represented by, e.g., the polarising beam splitters, the corner reflectors, semi-transparent mirrors and the demands for optical system adjustment. This is why using exclusively non-polarising interferometer with planar reflectors and a simple photodetector can be considered interesting direction towards cost-effective measurement systems, despite the necessity of laser beam modulation. Several extensions of the basic scheme of the homodyne detection are currently used in modern measurement systems, e.g., the use of planar mirrors [5] or advanced retroreflectors (MI-series interferometer, SIOS Messtechnik) instead of cube corner reflectors or the multichannel photodetector capable of producing the quadrature signals (Renishaw).

The next section presents the analytical description of the interference phenomenon and the signals produced by the quadrature detection unit. Note that the laser beam is considered as a set of monochromatic plane waves in vacuum since an ideal source of coherent light is assumed.

### 2.2. Quadrature Signals

The electromagnetic wave generated by a laser source can be described at a given point by a vector of electric field *E⃗* that oscillates in the plane perpendicular to the direction of propagation. If the direction of oscillations is fixed we call the wave linearly polarised and we can describe it by the magnitude of electric field *E*. For definiteness, assume that the laser source is placed at the origin of the Cartesian coordinate system and the wave propagates along the *x* axis.

The electric field at the source is given by the real part of the complex function

(2) $$E(t)=E_{0}\exp[\text{i}\phi (t)]$$

where *E*_0_ is amplitude and *φ*(*t*) is a general phase. By definition, the phase is related to the instantaneous angular frequency *w*(*t*) by

(3) $$w(t)=\frac{\text{d}\phi (t)}{\text{d}t}$$

Hence the phase can be calculated from the known angular frequency as

(4) $$\phi (t)=\int w(t)\text{d}t+\phi_{0}$$

The propagation of the wave from the source to the point *x* = *l* takes the time *τ* = *l*/*c*, where *c* is a velocity of propagation. Therefore at the point *x* = *l* the electric field is

(5) $$E(\tau ,t)=E_{0}\exp[\text{i}\phi (t-\tau )]$$

where *φ*(*t* − *τ*) is a phase shifted by time interval *τ*.

The intensity observed by a detector placed at the point *x* = *l* is proportional to the squared absolute value of electric field and is given by

(6) $$I(\tau ,t)=q{\left|E(\tau ,t)\right|}^{2}=qE(\tau ,t)E^{*}(\tau ,t)$$

where the asterisk denotes the complex conjugation and *q* is a proportionality constant.

Now assume there are two waves propagating along a single axis (practically representing two waves that travel along two arms of the interferometer). At the output of the interferometer the two waves are recombined, the interference occurs and the compound wave, usually denoted as an interference signal, is observed. Let *l*_1_ be the distance travelled from the source to the detector by the wave that propagates through the reference arm and *l*_2_ the distance through the measurement arm. Then the electric field of the compound wave at the detector is

(7) $$E_{1}(\tau_{1},\tau_{2},t)=E_{1}(\tau_{1},t)+E_{2}(\tau_{2},t)=E_{01}\exp[\text{i}\phi (t-\tau_{1})]+E_{02}\exp[\text{i}\phi (t-\tau_{2})]$$

where *τ*_1_ = *l*_1_/*c* and *τ*_2_ = *l*_2_/*c*.

The intensity at the point of observation can be calculated using Equation (6). Denoting $I_{1}=qE_{01}^{2}$ and $I_{2}=qE_{02}^{2}$ and using the Euler's identity for complex numbers we obtain the observed intensity

(10) $$I(\tau_{1},\tau_{2},t)=I_{1}+I_{2}+2\sqrt{I_{1}I_{2}}\cos[\phi (t-\tau_{1})-\phi (t-\tau_{2})]$$

Changing the variables *t* − *τ*_1_ → *t* and denoting *τ*_2_ − *τ*_1_ = *τ*, we rewrite the observed intensity as

(9) $$I(\tau ,t)=I_{1}+I_{2}+2\sqrt{I_{1}I_{2}}\cos[\phi (t)-\phi (t-\tau )]$$

We see that the observed intensity depends only on amplitudes of two interfering waves and their phase difference. The latter is caused by the path difference *l* = *l*_2_ − *l*_1_ between the paths travelled by two interfering waves.

When the laser source works on single frequency *f*, the angular frequency of the wave is constant and can be calculated as

(10) $$w=2\pi f=2\pi \frac{c}{\lambda }$$

where *λ* is wavelength. From Equation (4) we get the phase of the first wave

(11) $$\phi (t)=wt+\phi_{0}$$

where *φ*_0_ is the initial phase. Similarly, the shifted phase of the second wave is

(12) $$\phi (t-\tau )=w(t-\tau )+\phi_{0}$$

The intensity *I_x_* observed by the first branch of the detection unit can be calculated by means of Equation (9). We get

(13) $$I_{x}(\tau ,t)=I_{1}+I_{2}+2\sqrt{I_{1}I_{2}}\cos(w\tau )$$

which is a well-known result that allows one to observe interference fringes when the time delay *τ* = (*l*_2_ − *l*_1_)/*c* is not constant. This can be easily achieved by changing the length *l*_2_ of the measurement arm.

The intensity *I_y_* observed by the second branch of the detection unit (the Y-signal) that involves the retarder plate is shifted from *I_x_* by the phase factor *π*/2 and is given by

(14) $$I_{y}(\tau ,t)=I_{1}+I_{2}+2\sqrt{I_{1}I_{2}}\sin(w\tau )$$

On the basis of analytic model of the quadrature signals, the next subsection presents the approach to computational reconstruction of quadrature signals from a signal observed by simple detector.

### 2.3. Reconstruction of Quadrature Signals

As mentioned above, we employ a frequency modulated laser beam instead of a monochromatic one. Similar approach has been already employed, e.g., in phase-shifting and phase-modulation based interferometry [6–8], frequency modulated continuous wave interferometry [9] or for absolute distance measurement [4,10], nonetheless the follow-up evaluation is principally different from our approach.

Particularly, we apply the sinusoidal frequency modulation. First, we denote the mean value of angular frequency of the wave *w_s_* and the amplitude of the sinusoidal modulation *w_m_*. Then the angular frequency of the first wave is

(15) $$w(t)=w_{s}+w_{m}\sin(\Omega t)$$

where Ω is modulation angular frequency. The phase of the first wave can be obtained by integration of the angular frequency We get

(16) $$\phi (t)=\int w(t)\text{d}t+\phi_{0}=w_{s}t-\frac{w_{m}}{\Omega }\cos(\Omega t)+\phi_{0}$$

Similarly, the phase of the second wave shifted by the time delay *τ* is

(17) $$\phi (t-\tau )=w_{s}(t-\tau )-\frac{w_{m}}{\Omega }\cos(\Omega (t-\tau ))+\phi_{0}$$

The intensity *I_x_* observed by the first branch of the detection unit can be calculated using Equation (9). For clarity, we first calculate the phase difference *φ*(*t*) − *φ*(*t* − *τ*). Using the standard relations for trigonometric functions we obtain

(18) $$\phi (t)-\phi (t-\tau )=w_{s}\tau +\frac{2w_{m}}{\Omega }\sin\left[\Omega \left(t-\frac{\tau }{2}\right)\right]\sin\left(\frac{\Omega \tau }{2}\right)$$

This expression can be further simplified on the basis of the following considerations. Due to the typical dimensions of commonly used interferometers and a large value of the speed of light, the time delay *τ* is negligible compared to the time *t*. Moreover, the term Ω*τ* ≪ 1. Hence we can make the approximations

(19) $$\begin{array}{cc} \sin\left[\Omega \left(t-\frac{\tau }{2}\right)\right]≐\sin(\Omega t), & \sin\left(\frac{\Omega t}{2}\right)≐ \end{array}\frac{\Omega t}{2}$$

The phase difference is then

(20) $$\phi (t)-\phi (t-\tau )=w_{s}\tau +w_{m}\tau \sin(\Omega t)$$

Inserting this expression into Equation (9) we finally get the intensity *I_x_* observed by the first branch of the detection unit

(21) $$I_{x}(\tau ,t)=I_{1}+I_{2}+2\sqrt{I_{1}I_{2}}\cos[w_{s}\tau +w_{m}\tau \sin(\Omega t)]$$

The intensity *I_y_* observed by the second branch of the detection unit differs from *I_x_* only by global phase factor *π*/2, therefore we have

(22) $$I_{y}(\tau ,t)=I_{1}+I_{2}+2\sqrt{I_{1}I_{2}}\sin[w_{s}\tau +w_{m}\tau \sin(\Omega t)]$$

We see that the observed intensity is variable even if the time delay *τ* is constant. This is obviously caused by the modulation of the angular frequency of the wave emitted by the source.

The intensities *I_x_* and *I_y_* observed by particular branches of detection unit given by Equations (21) and (22) were derived for the general case of sinusoidal modulation of the angular frequency. In these calculations the depth of modulation *w_m_* and time delay *τ* were not subjected to any restrictions. However, we show that under certain assumptions for these two variables, the Equations (21) and (22) can be substantially simplified.

Since the absolute value *I*_1_ + *I*_2_ and the factor $2\sqrt{I_{1}I_{2}}$ are common for both *I_x_* and *I_y_*, we omit them for better readability of the following text. Mathematically, this corresponds to the normalisation of functions *I_x_* and *I_y_* by means of relations

(23) $$\begin{array}{cc} {\tilde{I}}_{x}=\frac{I_{x}-I_{1}-I_{2}}{2\sqrt{I_{1}I_{2}}}, & {\tilde{I}}_{y}=\frac{I_{y}-I_{1}-I_{2}}{2\sqrt{I_{1}I_{2}}} \end{array}$$

Then we start with normalised intensities

(24) $$\begin{array}{lll} {\tilde{I}}_{x}(\tau ,t) & = & \cos[w_{s}\tau +w_{m}\tau \sin(\Omega t)]\\ {\tilde{I}}_{y}(\tau ,t) & = & \sin[w_{s}\tau +w_{m}\tau \sin(\Omega t)] \end{array}$$

Using standard formulae for trigonometric functions we can rewrite them to

(25) $$\begin{array}{lll} {\tilde{I}}_{x}(\tau ,t) & = & \cos(w_{s}\tau )\cos[w_{m}\tau \sin(\Omega t)]-\sin(w_{s}\tau )\sin[w_{m}\tau \sin(\Omega t)]\\ {\tilde{I}}_{y}(\tau ,t) & = & \sin(w_{s}\tau )\cos[w_{m}\tau \sin(\Omega t)]+\cos(w_{s}\tau )\sin[w_{m}\tau \sin(\Omega t)] \end{array}$$

Since the maximum absolute value of the term sin(Ω*t*) equals to unity, assuming that *w_m_τ* ≪ l we can make the following approximations

(26) $$\begin{array}{lll} \cos[w_{m}\tau \sin(\Omega t)] & ≐ & 1,\\ \sin[w_{m}\tau \sin(\Omega t)] & ≐ & w_{m}\tau \sin(\Omega t) \end{array}$$

and for observed intensities we get formulae

(27) $$\begin{array}{lll} {\tilde{I}}_{x}(\tau ,t) & = & \cos(w_{s}\tau )-w_{m}\tau \sin(\Omega t)\sin(w_{s}\tau )\\ {\tilde{I}}_{y}(\tau ,t) & = & \cos(w_{s}\tau )+w_{m}\tau \sin(\Omega t)\cos(w_{s}\tau ) \end{array}$$

Both observed intensities *Ĩ_x_* and *Ĩ_y_* are now expressed as a sum of two terms. We explain the meaning of particular terms for intensity *Ĩ_x_*. The discussion for *Ĩ_y_* can be done in a similar way

The first term of *Ĩ_x_* expressed as cos(*w_s_τ*) depends only on the mean value of angular frequency *w_s_* and time delay *τ*. Hence it is identical to the corresponding term calculated for interference of two monochromatic waves, see Equation (13). The second term of *Ĩ_x_* is the product of amplitude *w_m_τ* sin(*w_s_τ*) and oscillating function sin(Ω*t*), which corresponds to the modulation of angular frequency. Since we have assumed that *w_m_τ* ≪ 1, the amplitude of the second term of *Ĩ_x_* is much smaller than the amplitude of the first term.

For further considerations we make the following assumption. When the mirror in measurement arm is moving we demand that the function sin(Ω*t*) oscillates much faster than cos(*w_s_τ*). Mathematically, this corresponds to the relation

(28) $$\frac{\text{d}(\Omega t)}{\text{d}t}\gg \frac{\text{d}(w_{s}\tau )}{\text{d}t}$$

For illustration, considering the motion of the mirror in measurement arm with a constant speed *v*, the last condition is expressed as Ω ≫ *w_s_v*/*c*.

The mean values 〈*Ĩ_x_*〉 and 〈*Ĩ_y_*〉 of observed intensities can be calculated using the relations

(29) $$\begin{array}{lll} \langle {\tilde{I}}_{x}(\tau ,t)\rangle & = & \frac{1}{T}\int_{0}^{T}{\tilde{I}}_{x}(\tau ,t)\text{d}t=\frac{1}{T}\int_{0}^{T}[\cos(w_{s}\tau )-w_{m}\tau \sin(\Omega t)\sin(w_{s}\tau )]\text{d}t\\ \langle {\tilde{I}}_{y}(\tau ,t)\rangle & = & \frac{1}{T}\int_{0}^{T}{\tilde{I}}_{y}(\tau ,t)\text{d}t=\frac{1}{T}\int_{0}^{T}[\sin(w_{s}\tau )+w_{m}\tau \sin(\Omega t)\cos(w_{s}\tau )]\text{d}t \end{array}$$

where *T* = 2*π*/Ω is a period of frequency modulation. The stated assumption Equation (28) means that the value of functions sin(*w_s_τ*) and cos(*w_s_τ*) can be considered constant in time interval from 0 to *T*. Moreover, since the integral of sine and cosine function over the period is zero, we get the mean values

(30) $$\begin{array}{lll} \langle {\tilde{I}}_{x}(\tau ,t)\rangle & = & \cos(w_{s}\tau ),\\ \langle {\tilde{I}}_{y}(\tau ,t)\rangle & = & \sin(w_{s}\tau ) \end{array}$$

We see that under the stated assumptions, the mean values of observed intensities when the frequency is modulated are identical to the intensities observed when the frequency is constant, see Equations (13) and (14). Note that the suitable depth of modulation is crucial for the reconstruction of the quadrature signals.

Now we would like to show the procedure that allows us to calculate the mean value of intensity 〈*Ĩ_y_*〉 from the observed intensity *Ĩ_x_*. We start with intensity *Ĩ_x_* at the time *t* given by

(31) $${\tilde{I}}_{x}(\tau ,t)=\cos(w_{s}\tau )-w_{m}\tau \sin(\Omega t)\sin(w_{s}\tau )$$

and its value at neighbouring time *t* − Δ*t*, which is

(32) $${\tilde{I}}_{x}(\tau ,t-\Delta t)=\cos(w_{s}\tau )-w_{m}\tau \sin(\Omega (t-\Delta t))\sin(w_{s}\tau )$$

Calculation of their difference Δ*Ĩ_x_* gives us

(33) $$\begin{array}{lll} {\tilde{I}}_{x}(\tau ,t) & = & {\tilde{I}}_{x}(\tau ,t)-{\tilde{I}}_{x}(\tau ,t-\Delta t)=\\ & = & w_{m}\tau \sin(w_{s}\tau )[\sin(\Omega (t-\Delta t)-\sin(\Omega t)]=\\ & = & -w_{m}\tau \sin(w_{s}\tau )[(1-\cos(\Omega \Delta t))\sin(\Omega t)+\sin(\Omega \Delta t)\cos(\Omega t)] \end{array}$$

Next, multiplying the difference Δ*Ĩ_x_* by cos(Ω*t*) (note that it is a phase-shifted copy of modulation signal, originally sin (Ω*t*)) and calculating the mean value we get the derived signal

(34) $$\begin{array}{lll} I_{d}(\tau ,t) & = & \langle \Delta {\tilde{I}}_{x}(\tau ,t)\cos(\Omega t)\rangle =\\ & = & -\frac{w_{m}\tau }{T}\sin(w_{s}\tau )\int_{0}^{T}[(1-\cos(\Omega \Delta t))\sin(\Omega t)\cos(\Omega t)+\sin(\Omega \Delta t)\cos^{2}(\Omega t)]\text{d}t\\ & = & -\frac{1}{2}w_{m}\tau \sin(\Omega \Delta t))\sin(w_{s}\tau ) \end{array}$$

Finally, from comparison with Equation (30) the derived signal *I_d_* is

(35) $$I_{d}(\tau ,t)=-\frac{1}{2}w_{m}\tau \sin(\Omega \Delta t)\langle {\tilde{I}}_{y}\rangle$$

We see that the derived signal *I_d_* is proportional to the mean value of intensity 〈*Ĩ_y_*〉. Hence, we are able to reconstruct the mean intensity 〈*Ĩ_y_*〉 from intensity *Ĩ_x_*.

The last part of the novel detection method, presented in the final part of this section, addresses the issues with the practical implementation.

### 2.4. Scale Linearity Compensation

We have identified several sources of inaccuracies related to a practical implementation of the proposed method:

- the measured intensities *I_x_* and *I_y_* are generally burdened with a measurement error, e.g., noise or the quantisation error (*i.e.*, the error caused by the discretization of analog measure),
- the currently available laser sources are liable to a residual amplitude modulation (RAM) [11], *i.e.*, the modulation of their optical frequency has an unwanted influence on the intensity and spatial characteristics,
- the computation over discrete representation of the analog signals causes additional inaccuracy, e.g., numeric calculation of derivation, round-off error.

Note that the list of influences is far from being complete, nonetheless these were the most significant ones that we met and found important to be addressed.

Currently, the problems with scale linearity (i) are well explored [12,13] and there are several methods to deal with them [14–16].

The RAM effect (ii) is superimposed onto the effect of the laser frequency modulation. As a consequence, there occurs an additional phase shift between *I_x_* and *I_y_* besides the desired *π*/2 when our detection method is used. This results in an unwanted offset in the detected interference phase that depends on the difference of path-lengths and the modulation depth. The effect is additionally amplified due to the discrete computation (iii), especially due to the calculation of the numeric difference over a discrete set of sampled points.

The phase shift is typically corrected by the scale linearization techniques, nonetheless our preliminary experimentation indicated that a compensation of the phase shift between the quadrature signals before the elliptic-fitting based linearization technique leads to better overall performance of our method. On the basis of this experience, we have employed both techniques: the phase shift removal is described in the following section and for the scale linearization technique we used an existing method [14]—the latter method is not described in this article.

### 2.5. Removal of Unwanted Phase Shift between Quadrature Signals

The principle of our phase shift removal method is the following: after calculation of *I_y_* from the observed *I_x_* we detect their mutual phase shift *δ_a_* (caused mainly by RAM), then we transform *I_y_* in an appropriate manner to correct this phase shift and after that we use the linearization method.

The following description presents the method for a general case, *i.e.*, we assume unequal amplitudes of the quadrature signals, even though we have operated with unit amplitudes of *I_x_* and *I_y_* before.

We start again with intensities *I_x_* and *I_y_* in the form

(36) $$\begin{array}{lll} I_{x} & = & K_{x}\cos\phi ,\\ I_{y} & = & K_{y}\sin(\phi +\delta_{a})=K_{y}\cos\left(\phi +\delta_{a}-\frac{\pi }{2}\right) \end{array}$$

where we assume that their mean value is zero and *K_x_, K_y_* are real amplitudes of *I_x_, I_y_*. Note that the amplitudes are known from the observed time course of *I_x_* and *I_y_* in practical applications. Next, we introduce a quantity *K_xy_* that corresponds to the amplitude of the sum *I_x_* + *I_y_*, formally written as

(37) $$K_{xy}=\max(I_{x}+I_{y}).$$

The value *K_xy_* is also known from observed data.

**Figure 2.**
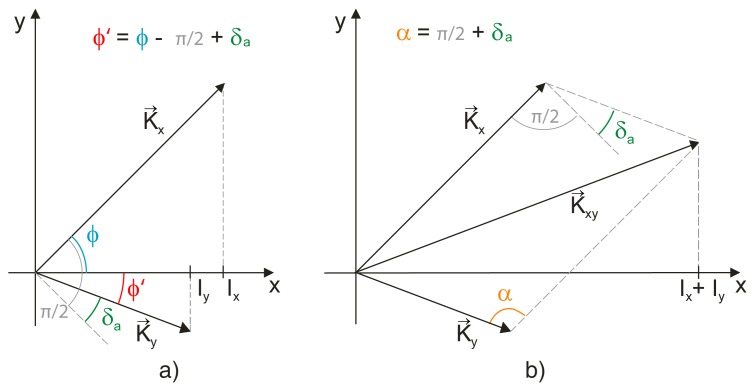
Detection of the unwanted phase shift *δ_a_*: the known instantaneous values *I_x_, I_y_* are projected to phasors *K⃗_x_, K⃗_y_* that form the angles *φ* (equal to the immediate interference phase) and *φ*′ = *φ* − *π*/2 + *δ_a_* with axis *x* (**a**); a combination of *K⃗_x_* and *K⃗_y_* forms phasor *K⃗_xy_* (**b**). Since there exists such *φ* such that *K⃗_xy_* is parallel to axis *x*, the real angle *α* = *π*/2 + *δ_a_* can be calculated using law of cosines.

We illustrate the functions *I_x_* and *I_y_* given by Equation (36) in a phasor diagram using two phasors *K⃗_x_* and *K⃗_y_* of lengths *K_x_* and *K_y_*, respectively (see Figure 2(a)). These phasors together form a constant angle *π*/2 − *δ_a_* resulting from Equation (36), but they both rotate around the origin as the phase *φ* changes. In such a diagram the values *I_x_* and *I_y_* are simply given by orthogonal projection of the phasors *K⃗_x_* and *K⃗_y_* to the x-axis, respectively.

The geometrical meaning of quantity *K_xy_* is the following: since it was denned as the amplitude of sum *I_x_* + *I_y_*, it represents a length of phasor *K⃗_xy_* = *K⃗_x_* + *K⃗_y_* in the phasor diagram, see Figure 2(b). To derive the expression between the lengths *K_x_, K_y_, K_xy_* and phase difference *δ_a_*, we use the law of cosines. Since the angle *a* shown in Figure 2(b) equals to *δ_a_* + *π*/2, we get

(38) $$K_{xy}^{2}=K_{x}^{2}+K_{y}^{2}-2K_{x}K_{y}\cos\left(\delta_{a}+\frac{\pi }{2}\right)$$

It should be emphasised that the function *K_xy_*(*δ_a_*) is monotonic in the range − *π*/2 ≤ *δ_a_* ≤ *π*/2 for arbitrary values of *K_x_* and *K_y_*, which can be easily verified by calculating the derivative of *K_xy_* with respect to *δ_a_*. This allows us to solve Equation (38) with respect to *δ_a_*. We obtain

(39) $$\delta_{a}=\arcsin\left(\frac{K_{xy}^{2}-K_{x}^{2}-K_{y}^{2}}{2K_{x}K_{y}}\right)$$

Since the value *K_xy_* and amplitudes *K_x_, K_y_* can be easily determined from measured data, we are able to calculate the phase shift *δ_a_* and eliminate the unwanted phase shift between the quadrature signals.

For this purpose we now need to transform intensities *I_x_, I_y_* given by Equation (36) to another pair $I_{x}^{\prime }$, $I_{y}^{\prime }$ given by

(40) $$\begin{array}{lll} I_{x}^{\prime } & = & K_{x}\cos\phi ,\\ I_{y}^{\prime } & = & K_{y}\cos\phi . \end{array}$$

Here $I_{x}^{\prime }$ is chosen to be identical to *I_x_*, for $I_{y}^{\prime }$ we need to derive the transformation relation expressed in terms of *I_x_, I_y_* and *δ_a_*. Therefore we rewrite Equation (36) as

(41) $$\begin{array}{lll} I_{x} & = & K_{x}\cos\phi ,\\ I_{y} & = & K_{y}(\sin\phi \cos\delta_{a}+\cos\phi \sin\delta_{a})=I_{y}^{\prime }\cos\delta_{a}+I_{x}\frac{K_{y}}{K_{x}}\sin\delta_{a} \end{array}$$

Solving the second equation with respect to $I_{y}^{\prime }$ we obtain the transformation relations

(42) $$\begin{array}{lll} I_{x}^{\prime } & = & I_{x}\\ I_{y}^{\prime } & = & \frac{I_{y}K_{x}-I_{x}K_{y}\sin\delta_{a}}{K_{x}\cos\delta_{a}} \end{array}$$

The transformed intensities $I_{x}^{\prime }$ and $I_{y}^{\prime }$ are now deprived from the additional phase shift *δ_a_* caused by amplitude instability of the laser source. As a next step, the signals are scaled to unit amplitude, the scale linearization is applied and then the phase is extracted.

Note that the algorithm is principally based on a single data-point determined from the measured data (maximum value of *I_x_, I_y_*, …), so that some outlying data points might cause a substantial error in the calculations. Since the *K_x_* and *K_y_* (and *δ_a_* as well) can be considered constant, the method can rely on some kind of statistical information (and actually has within our experiments).

## 3. Experimental Verification

We have carried out several experiments to verify the performance of the proposed technique. We have assembled an optical setup that allowed us to make the displacement measurements at the nanometre scale referenced to an interferometer with the homodyne detection technique, *i.e.*, quadrature detection unit.

The reference was physically provided by a calibrated quadrature detection unit in combination with the support logic. The reference delivers the reference phase information with measurement uncertainty of *N/*1,024 (where *N* denotes single interference fringe), *i.e.*, 0.35° [17] (including the linearity compensation).

### 3.1. Experimental Setup

**Figure 3.**
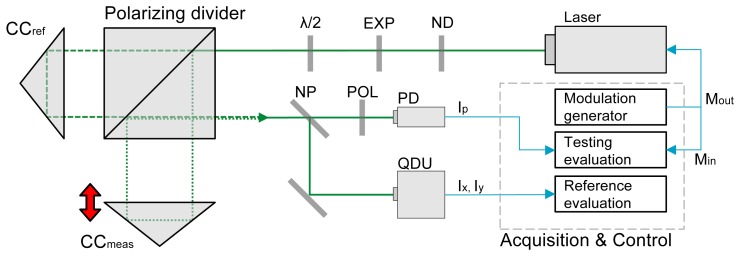
Experimental setup scheme: the interferometer employs the polarising divider, the reference corner cube CC*_ref_* and measurement corner cube CC*_meas_*. The laser input is attenuated by a neutral density absorption niters ND, collimated by an optical expander EXP and its polarisation plane is rotated by the half-wave plate *λ*/2. The interferometer output is split into two detection branches by the non-polarising divider NP In the first branch, the interference occurs on the polariser POL and the intensity is observed by a photodetector PD. In the second branch, the signal from the interferometer is processed by the reference quadrature detection unit QDU. The acquisition and control hardware drives the modulation of the laser source optical frequency and evaluates the signals from both the photodetector and the quadrature detection unit.

The experimental setup, schematically shown in Figure 3, comprised a Michelson interferometer with polarising optics and two detection branches: the reference branch with a quadrature detection unit and the testing branch that performed the proposed technique for interference phase detection.

A solid-state laser (frequency doubled Nd:YAG at 532 nm with linearly polarised beam; PROMETHEUS by Innolight, Germany) was used. The output beam was collimated to particular diameter (3 mm) that optimally fits the detection unit and the photodetector. A half-wave plate (*λ*/2) was used to adjust the polarisation plane. The measuring reflector was mounted on a piezo actuator and on a motorised stage. This combination allowed for the measurement on an operational range of 600 mm. Note that the polarising variant of the beam splitter is used, as it is a necessary condition for the reference interferometer (the detection apparatus, not to be confused with the reference arm of the interferometer). The proposed method does not require preserving different polarisation in individual arms.

A non-polarising splitter (NP) is used to form two detection branches: the testing branch that involves our novel detection method with an ordinary photodetector (PD) and the reference one that uses the traditional detection unit (denoted QDU).

The bandwidth of both detection methods is limited by the combination of several factors: response of the photodetector, signal amplifiers, analog-to-digital conversion sample rate and processing capability. In our particular case, the available bandwidth was 50 kHz for the QDU and 100 Hz for the novel method (*i.e.*, 100 phase readings by QDU and 200 phase readings by tested method per second). The optical line-up in the testing branch is extended by a polariser plate that alters the orthogonal polarisations into single plane so that testing branch observes the interference signal instead of compound wave (that is processed by the reference branch). The bandwidth of our novel method is relatively low, nonetheless the sense was to demonstrate the principle and compare the linearity with a reference method.

The signal generation (for laser source modulation) and acquisition was done by a dedicated DAQ device (U2531 by Agilent). The generation part produced synchronised sine and cosine wave—the sine controlled the modulation, the cosine was synchronously sampled (acquired) together with the output of the QDU and the PD output and used for the quadrature signal reconstruction. The reason to generate the modulation signal and its phase shifted copy and then sample the copy again was to achieve precise synchronisation between the generated outputs and signal inputs.

### 3.2. Design of Experiments

The measurement arm hardware was successively placed into different positions (measurement points)

ΔL∈{0,1,2,5,5,7.5,10,15,25,37.5,25,37.5,50,62.5,75,100,150,200,250,300,500,600}mm

to cover the operating range Δ*L* ∈ [0, 600] mm. At each point, the measurement arm was continuously displaced (with approximately constant velocity using the piezo actuator) over the trajectory of ≈0.6 *μ*m(more than two complete fringes) and the detection outputs were acquired. The selection of points was suggested by preliminary experiments that indicated that the correct distance determination is more problematic at the beginning of the operating range. The zero position (Δ*L* = 0) actually denotes a closest achievable position of the measurement arm, given by the mechanical construction of interferometer components and corresponds actually to the path length difference of approximately 2 mm. The laser source has been modulated by a free running sine wave (at 1 kHz). The depth of modulation was empirically estimated and manually configured for each point—the optical frequency was tuned approximately by ±20…50 MHz.

The outputs from the quadrature detection unit *I_x_, I_y_*, the photodetector signal *I_p_* and modulation signal *M_in_* were synchronously acquired (at sample rate of 100 kHz). One phase reading of the novel method took a time period that corresponds to five complete modulation periods (5 ms). Three measurement cycles were recorded at each measurement point. The evaluation was performed afterwards over the entire cycles at once.

We have inspected the difference between the interference phase information extracted by the reference and by the testing method, referred to as the phase determination error (PDE). In the reference branch, the X- and Y-axis signals from the quadrature detection unit were recorded and the scale linearization was applied. The photodetector output *I_p_* was used for reconstruction (standing for the *I_x_* when related to the previous analytic description) of the quadrature signals using the presented method.

## 4. Results and Discussion

### 4.1. Results

**Figure 4.**
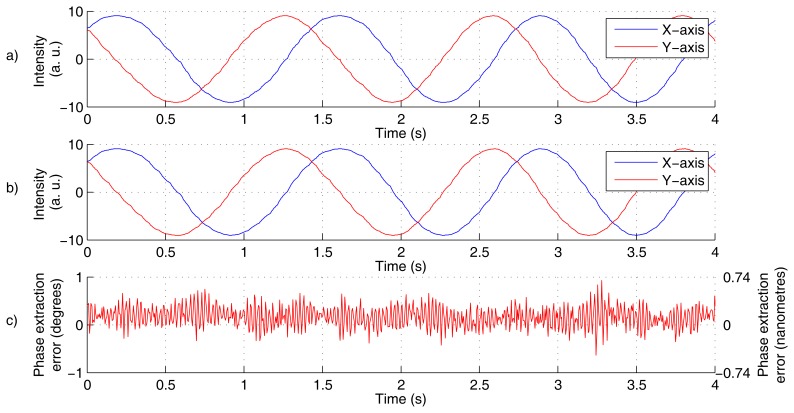
Evaluation of single experimental cycle: the X- and Y-axis signals from quadrature detection unit, *i.e.*, the 〈*Ĩ_x_*(*τ*, *t*)〉 and 〈*Ĩ_y_*(*τ*, *t*)〉 (refer to Equation (30)), with scale linearity compensation applied (**a**); X- and Y-axis signals reconstructed by proposed method, *i.e.*, 〈*Ĩ_x_*(*τ*, *t*)〉 and *I_d_* (Equation (35)), reconstructed from the signal observed by the photodetector with the phase shift removal and scale linearization applied (**b**) and the phase determination error, PDE (**c**).

A total of 60 measurement cycles in 20 positions was completed, where each cycle contains 890 phase readings. A sample evaluation of a single cycle is shown in Figure 4.

There is an apparently good coincidence between the quadrature signals from reference and testing detection branch (compare Figure 4(a) to Figure 4(b)) (the slight fluctuations are caused by changes in refractive index of air, but since the optical paths are shared, the influence is subtracted within the comparison). The phase extraction error plot (see Figure 4(c)) indicates that the PDE keeps within ±1° range (for the particular sample).

**Figure 5.**
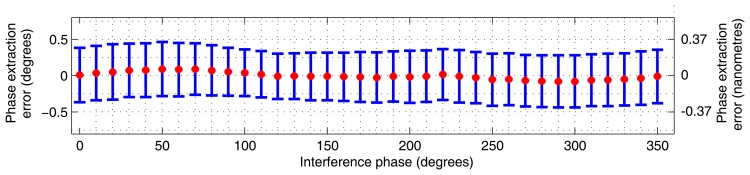
Experimental results: the red points show the periodic error, *i.e.*, the dependence of the mean phase determination error on immediate interference phase; the blue error bars indicate the interval of radius of standard deviation around the mean PDE at each corresponding point *i* (*n_i_* ≈ 1,485)

**Figure 6.**
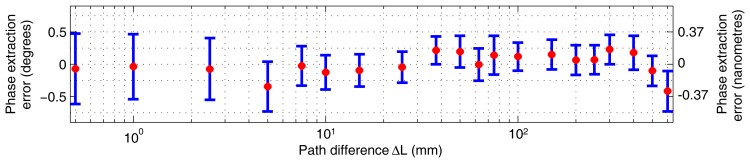
Experimental results: the red points show the dependence of the mean phase determination error on the path length difference; the blue error bars indicate the interval of radius of standard deviation around the mean PDE at each corresponding point *i* (*n_i_* ≈ 2,670)

The linearity of our method is summarised in Figure 5. There is an observable periodic error, and the mean PDE fluctuates within ±0.15°. The Figure 6 shows the overall dependence of the mean PDE on the path length difference when the results from all three cycles measured at the same distance point were combined so that the repeatability aspect is incorporated. The overall mean PDE stays below ±0.5° and the standard deviation stays below ±0.75°. The jitter of the mean error over the operational distance can be attributed to the optical adjustment. The combination caused a slightly greater scatter of the phase determination error.

Several related issues were omitted within the experimentation, e.g., the refraction index of air influence [18], the uncertainty of the laser source stability or a precise temperature stabilisation. In our case, when two detector paths were compared, these influences were suppressed by the differential measurement, since the optical paths and signal acquisition within the detection chain were common for both methods. The results show that the novel method has a comparable accuracy to the reference method. Exploring the limits of our proposed method is still a subject to a future research and a comparison with a more accurate method is required to state the absolute precision and uncertainty.

### 4.2. Discussion

The results indicate that the proposed method is a suitable alternative to the traditional homodyne detection, especially in situation when the accuracy is not the most critical aim but the construction simplicity and scalability is. There are several issues that need to be considered to judge the novel approach: its dynamic range, the trade-off between optical complexity and the requirement for laser modulation, the response bandwidth limitations and the uncertainty introduced by the computations.

As the homodyne detection has theoretically infinite dynamic range, it allows for the measurement with the same precision even on long distances (several kilometres). The practical limitation is given only by the construction issues, laser stability and influences of the environment. The novel method is principally limited by the available depth of laser frequency modulation [19], but using an approach for an adaptive control of the modulation depth the method would be probably applicable on a comparable operational range.

There are also higher demands posed on the laser source—there is the need for the laser beam frequency modulation. The modulation bandwidth limits the response of the measurement systems since the duration of at least one complete modulation period (five in case of our experiments) is processed to produce a single phase reading. Consequently, velocity of measurement is significantly slower than the optical processing. The demand for laser with a fast modulation capability can be fulfilled, to a certain extent, by cost-effective semiconductor lasers [20,21].

On the other hand, the method requires less complex optical setup to achieve comparable results—there is no need for polarising beam splitters and semi-transparent mirrors within the optical lineup. As a consequence, the method also scales well, e.g., when several measurement axes would be required within a measurement system. In such a case, the added complexity per each added axis is minimal in comparison to the traditional means—it has been already demonstrated in a practical application for surface diagnostics [22] and multi-axis geometric measurements [23]. An extra axis requires only a small amount of additional optical components and poses no substantial additional requirement on the laser source (assuming there is enough power available, but it is usually not an issue, e.g., for green Nd:YAG lasers).

In our experiments, the modulation bandwidth was limited by available high-voltage amplifiers to 200 phase readings per seconds (corresponds to 1 kHz modulation). With the particular laser source, a 100 × higher response would be achievable; further enhancement would be possible using and acousto-optic modulator.

In the basic principle, similar computational methods are also sensitive to the noise issues. In case of our novel method, the issue is well mitigated by a massive averaging and suitable signal filtering without increased demands on system performance as the operations are essential parts of the phase extraction method.

There were also several assumptions we stated (in sake of simplicity) in the physical description of the novel approach. We assume the time delay *τ*—the difference in wave propagation along unequal optical paths in the arms of the interferometer—is negligible (see the paragraph above Equation (19)). In the borderline case, the path length difference *l* = 1,200 mm, so the time delay *τ* ≈ 4 × 10^−9^ and the term fir Ω*τ* ≈ 4 × 10^−6^. We also assume that the product of *τ* and the modulation depth *w_m_τ* ≪ 1 (Equation (26)). In the case when *l* = 1,200 mm and *w_m_* = 20 MHz, the product *w_m_τ* = 0.08, which corresponds to the limit of validity of our assumption *w_m_τ* ≪ 1. Finally, we assume that the phase change (due to displacement) is slower than the modulation of the laser, *i.e.*, Ω ≫ *w_s_v*/*c* (Equation (28)). The displacement velocity *v* was *v* ≈ 2.5 × 10^−7^m/s, the Ω = 1kHz, thus for the mean frequency *w_s_* ≈ 563.25 THz the ratio is 1 × 10^3^ ≫ 7.5 × 10^−6^.

## 5. Conclusions

We have proposed and verified a novel detection technique that represents a novel approach to the homodyne detection in laser interferometry.

A detailed analytic description of the principles has been presented and the validity and performance were evaluated by experimental means.

The experimental results indicate that the novel approach performs comparably to the traditional detection technique. Since it requires simpler optical hardware setup—e.g., exclusively a non-polarising interferometer, single photodetector, but frequency modulated laser beam and computational power—it can be considered a suitable alternative to the techniques that rely on additional optical processing.
